# Prevent 6:1 trial: pI rotocol for a feasibility trial of a 6:1 intermittent diet for weight gain prevention in women at increased risk of breast cancer

**DOI:** 10.1136/bmjopen-2025-113204

**Published:** 2026-04-29

**Authors:** Laura Kinsey, David French, Sacha Howell, Jason Gill, Cheryl Lombardelli, Mai Haiba, Emma Barrett, Natasha Jones, Julie Wisely, Michelle Harvie

**Affiliations:** 1 Manchester Adult Cystic Fibrosis Centre, Wythenshawe Hospital, Manchester University NHS Foundation Trust, Manchester, UK; 2 The Prevent Breast Cancer Research Unit, Manchester University NHS Foundation Trust, Manchester, UK; 3 Division of Psychology and Mental Health, School of Health Sciences, The University of Manchester Faculty of Biology Medicine and Health, Manchester, UK; 4 Division of Cancer Sciences, The University of Manchester, Manchester, UK; 5 BHF Glasgow Cardiovascular Research Centre, School of Cardiovascular & Metabolic Health, University of Glasgow, Glasgow, UK; 6 Manchester Academic Health Science Centre, Manchester, UK; 7 Department of Medical Statistics, Manchester University NHS Foundation Trust, Manchester, UK; 8 Research and Innovation, Manchester University NHS Foundation Trust, Manchester, UK; 9 Greater Manchester Mental Health NHS Foundation Trust, Manchester, UK

**Keywords:** Weight Gain, Breast tumours, Fasting, Overweight, Primary Prevention, NUTRITION & DIETETICS

## Abstract

**Introduction:**

Weight gain in adulthood is a common potentially modifiable breast cancer risk factor. Intermittent 5:2 diets (two low-calorie days/week) have proven efficacy for weight loss among people with overweight or obesity and can promote sustained awareness and mindfulness of diet choice and help appetite control.

This trial aims to test whether a less intensive 6:1 intermittent diet programme (one low-calorie day/week) is a feasible intervention to promote healthy eating and prevent weight gain in women at increased risk of breast cancer.

**Methods and analysis:**

Single arm prospective feasibility trial in 30 healthy weight women aged 18–40 years, at moderate or high risk of breast cancer (≥17% lifetime risk and/or ≥3% 10 year risk at 40 years), body mass index 20–25 kg/m^2^. Participants will be entered to a 16-week 6:1 diet programme involving 1 day consuming 1000 kcal and healthy eating for 6 days a week. Participants will also be advised to meet physical activity recommendations for health (≥150 min of moderate intensity physical activity/week and resistance exercise two times per week). The programme will be supported remotely by dietitian calls at baseline, week 1, 4, 8, 12 and 16. Participants will also be provided access to a trial-specific private monitored Facebook group which includes information and the opportunity for peer support.

Co-primary outcomes are: (a) uptake to the trial, (b) retention rate, (c) adherence to the 6:1 diet and (d) participant feedback on acceptability of the programme. Secondary outcomes include characteristics of those recruited and completing the programme and a preliminary evaluation of benefits and harms. This includes changes in body weight and body composition (bioelectrical impedance), diet quality, physical activity, binge eating, sleep quality (Pittsburgh Sleep Quality Index), menstrual cycle length and potentially diet-related adverse events, that is, fatigue, constipation, dizziness, headache, indigestion. Exploratory outcomes include the impact of low-calorie days on dietary intake and physical activity across the week and any differences in adherence to the low calorie days across the menstrual cycle.

**Ethics and dissemination:**

This trial has been approved by South Central—Berkshire B Research Ethics committee (rec reference 24/SC/0321). Findings will be disseminated via peer-reviewed journals, national and international cancer prevention and obesity conferences and cancer prevention charitable bodies.

**Trial registration number:**

ISRCTN14330469.

**STRENGTHS AND LIMITATIONS OF THIS STUDY**

- The trial has clear progression criterion to decide whether to advance to a randomised controlled efficacy trial.
- This feasibility trial is not powered or of sufficient duration to demonstrate changes in weight or diet or physical activity behaviours.
- Women joining this trial are likely to be highly motivated and adherence may not reflect that seen in the wider population.
- The trial includes a quantitative evaluation but does not include a qualitative evaluation of acceptability of the programme.

## Introduction

 Adult weight gain is a potentially modifiable risk factor for developing breast cancer as well as nine other weight-related cancers in women.^1^ In the UK, 35% of women are a healthy weight with 62% overweight or obese.^2^ On average, women in the UK gain 10 kg of weight during adult life, mostly between the age of 18 and 40 years.^3^ This level of weight gain increases weight-related cancers in women by 20%, specifically increased rates of breast (15%), endometrium (50%) and kidney cancer (30%).^1^

Weight gain is a consequence of habitual chronic positive energy balance, largely driven by excess energy intake and often high fat, high sugar diets.^3^ Once weight is gained, it is difficult to lose and keep off for both physiological and behavioural reasons.^4^ Weight gain is common among women attending family history clinics (FHCs) who are at increased genetic risk of breast cancer. Weight gain has an equal or greater effect on the relative risk for breast cancer among women with a family history of breast cancer compared with those without a family history.^5^ Thus, there is an unmet need for effective diet and physical activity interventions to prevent weight gain and manage breast cancer risk in this population.

Our previous research has demonstrated that the 5:2 diet, involving 2 days in the week with restricted energy intake (800–1000 kcal/day), is efficacious for weight loss in people with overweight or obesity,^6^ and this approach has been successfully implemented in many healthcare settings in the UK and worldwide including patients with cancer^7^ and type 2 diabetes.^8^ Our research has shown that intermittent diets (such as the 5:2 diet) can promote sustained awareness and mindfulness of diet choice and helps to control appetite, which in turn promote maintained lower energy intakes across the week.^9^ It is likely that the intensity of dietary restriction needed for the prevention of weight gain is less than that needed to induce weight loss. The present trial will therefore test whether a less intensive 6:1 intermittent diet including one low-calorie day/week is acceptable and has the potential to promote a healthy diet to prevent weight gain in women at increased risk of breast cancer who are a healthy weight.

The trial will also assess if the diet could be associated with any harms. We will assess the effects of the diet on disordered eating. Intermittent weight loss diets have mainly demonstrated improvements in eating patterns in the weight loss setting,^10 11^ but some concerns have been expressed of increased eating related thoughts, hunger and fear of loss of control among healthy-weight individuals consuming such diets.^12 13^ There is also evidence that reduced calorie intakes may be associated with reduced physical activity and reduced energy expenditure, in order to conserve energy, with potential detrimental effects on lean body mass.^14^ Some people following energy restricted diets report poorer sleep quality,^15^ although findings are not consistent.^16^ The planned trial therefore includes assessments of dietary intake, an objective measure of physical activity (ACTi graph),^17^ sleep quality (Pittsburgh Sleep Quality Index (PSQI)) and body composition (bioelectrical impedance, Tanita MC980MA), waist, hip circumferences to obtain preliminary data of these effects.

As there is a potentially bi-directional relationship between low-calorie diets and menstrual cycle in young women, we will ask participants to record the first day of their menstrual cycle throughout the trial. This is being done for completeness as we do not anticipate the modest energy restriction with the 6:1 intermittent diet will directly affect menstrual cycle function which can occur with longer spells of low-calorie intake.^18^ Inclusion of menstrual cycle dates will also provide preliminary data of any potential differences in dietary adherence across the menstrual cycle and whether adherence is reduced in the premenstrual weeks when women often report increased appetite and carbohydrate craving.^19^

In line with guidance on best practice in evaluating complex interventions, it is important to test the acceptability and any potential benefits and harms of this novel diet in a feasibility trial, as well as assess the major uncertainties in conducting an efficacy trial of this diet.^20^ The evaluation of acceptability, adherence and exploratory evaluation of weight and health behaviours and patient experience in the trial will determine whether we should proceed to a larger efficacy trial.

### Aim

To test whether a 6:1 intermittent diet programme (one low-calorie day/week) is an acceptable intervention and has the potential to promote healthy eating and prevent weight gain in women at increased risk of breast cancer.

## Methods and analysis

### Trial design

This trial is a single arm prospective feasibility trial among women aged 18–40 years, at moderate or high risk of breast cancer (>17% lifetime risk and/or with 10-year risk of breast cancer of >3% at age 40 years)^21^ with a body mass index (BMI) ≥20 and ≤25 kg/m^2^.

### Trial setting and recruitment

Participants will be identified from the regional FHC and the Cancer Genetics Clinics at Manchester University NHS Foundation Trust (MFT). Recruitment will be through direct invitation by members of the clinical teams, mailing the patient information sheet and promotion via the Prevent Breast Cancer charity website and related social media platforms such as Facebook and X. These women will have their attendance at the FHC and genetics clinics verified by a member of the relevant teams. The planned trial start and end dates are 17 March 2025 and 17 March 2026. All trial face-to-face assessments will be undertaken by the research team in a research facility at the main recruiting site at Wythenshawe Hospital. All participants will have a trial run-in week before they receive baseline diet advice when we ask them to record 7 days of habitual diet and wear an activity monitor to assess their habitual diet and physical activity patterns.

### Eligibility criteria

Interested participants will be asked to contact the 6:1 trial research team who will provide more information about the trial and complete preliminary screening questions using inclusion and exclusion criteria. Ineligible people will be asked if we can keep their eligibility responses and record their verbal consent so we can report uptake to the trial and reasons for screen fail. Potential participants who express interest in participating and who appear to meet the inclusion criteria will be invited to a screening and baseline visit where they will be asked to provide written informed consent to enter the trial (online supplemental file 1). Full eligibility for the trial will be checked at the face-to-face baseline visit and includes; current weight, height, BMI, pregnancy status, Binge Eating Scores (BESs) <27 (range 0–62),^22^ Generalised Anxiety Disorder (GAD-7) <15 (range 0–21),^23^ depression scores using Patient Health Questionnaire-9 (PHQ-9) <15 (range 0–27)^24^ and Alcohol Use Disorders Identification Test (AUDIT) >16 (range 0–40). Full inclusion and exclusion criteria for the trial are reported in box 1.

Box 1 **Inclusion and exclusion criteria for the Prevent 6:1 trial**

**Inclusion criteria**

- Aged 18–40 years.
- Born female and not undergone gender reassignment X.
- Moderate or high risk of breast cancer (≥17% lifetime risk or with 10-year risk of breast cancer of ≥3% at 40)^21^ and have attended/currently attending NHS family history/genetics clinic.
- BMI 20–25 kg/m^2^.
- Able to communicate (written and spoken) in English or any other language and access to a phone as we have access to an interpreter service.
- Able to attend two face-to-face appointments at MFT at baseline and week 16.
- Not pregnant or planning to become pregnant in the next 16 weeks. Where appropriate, participants will need to have negative urine pregnancy test at screening and agreement to maintain contraception or abstinence during the trial.

**Exclusion criteria**

- Previous breast cancer or bilateral preventative mastectomy.
- Currently trying to gain weight.
- Previously had weight loss surgery or taking weight loss medication.
- Have a medical condition that influences diet and weight, for example, diabetes, inflammatory bowel disease or cystic fibrosis.
- Current diagnosis of a psychiatric disorder, that is, bipolar psychotic disorder or current self-harm.
- Current or previous diagnosis of an eating disorder.
- Substance abuse or harmful alcohol use as indicated by a score of 16 or above on the AUDIT Test.
- Severe binge eating assessed by a score of 27 or more on the BES.
- Severe depression assessed by a score of 15 or more on the PHQ-9.
- Severe anxiety assessed by a score of 15 or more on the GAD-7 questionnaire.
- Confirmed pregnancy via a pregnancy test, planning pregnancy in the next 16 weeks, or currently breastfeeding.

AUDIT, Alcohol Use Disorders Identification Test; BES, Binge Eating Scale30; BMI, body mass index; GAD-7=General Anxiety Disorder-7; MFT, Manchester University Foundation Trust; PHQ-9, Patient Health Questionnaire-9. NHS, National Heath Service.

### Sample size

We plan to recruit 30 participants which provides an acceptable degree of precision for estimates for the three co-primary outcomes of the trial: uptake, retention and dietary adherence. Using a 90% CI, a sample size of 30 provides estimates of trial uptake of 10% with an error of ±9%, trial retention at 16 weeks of 50% with an error of ±15% and adherence to the 6:1 intermittent diet (ie, the number of weeks which included one low calorie/day versus the number of weeks on the trial 40% with an error of ±15%.

Previous diet studies in this population have had an uptake of around 15%.^25^ To recruit 30 women, we predict we will need to invite ~200 women. There are currently ~2000 women aged 18–40, with BMI 20–25 kg/m^2^ within the regional FHC at MFT. We anticipate recruitment will take place over 12–20 weeks.

### The 6.1 diet programme

#### Dietary advice

Participants will receive advice on adopting an intermittent 6:1 diet which involves one 1000 kcal day/week including ~70 g of protein and ~100 g carbohydrate. The 1000 kcal diet comprises 6–7 portions ~210 g of lean protein containing foods, 3–4 portions of wholegrain carbohydrates, one 7 g portion of fat, five 80 g portions of vegetables, two portions of fruit and three portions of dairy or dairy alternatives. An example of a low-calorie day is provided in table 1. The diet also includes 6 days/week of healthy eating Mediterranean-type diet which provides approximately 30% energy from fat (15% monounsaturated fatty acids, 8% polyunsaturated fatty acids, 7% saturated fatty acids), 25% energy from protein and 45% from low glycaemic load carbohydrate. The diet includes at least five portions of vegetables and two portions of fruit/day and is limited in alcohol (<10 units, 80 g /week) as described previously.^6^ Food and drink will be self-selected and not provided by the trial team. The diet can be successfully adapted for people of different ethnicities and those following omnivorous, vegetarian and vegan diets.

**Table 1:** Example of a low calorie day for a mixed omnivorous diet which includes meat, fish or dairy

	Portion	Numbers of the food portions/day					
		Dairy	Protein	Carb	Veg	Fruit	Fat
*Breakfast*
Poached egg	1 egg	0	1	0	0	0	0
Grilled tomatoes	7 cherry tomatoes	0	0	0	1	0	0
Tea/coffee	1 mug	0	0	0	0	0	0
*Midmorning*							
Natural yoghurt ½ handful of berries	150 g 40 g	1	0	0	0	1/2	0
*Lunch*							
Wholegrain bread	1 medium slice	0	0	2	0	0	0
Tuna	⅓ of a 120 g can	0	1	0	0	0	0
Green salad	Cereal bowl (80 g) with oil-free dressing	0	0	0	1	0	0
Satsuma	1	0	0	0	0	1/2	0
*Mid afternoon*							
Low fat cheese	30 g /match box size	1	0	0	0	0	0
Apple slices	I medium apple (80 g)	0	0	0	0	1	0
Tea/coffee		0	0	0	0	0	0
*Evening*							
Vegetable rice	4 tablespoons cooked rice and 160 g of mixed vegetables	0	0	2	2	0	0
Chicken curry	90 g/average chicken breast (no skin) and ½ can tomatoes, 1 dessert spoon of oil	0	3	0	1	0	1
*Bedtime*							
Low fat houmous	1 level tablespoon	0	1	0	0	0	0
Pepper sticks	½ red pepper	0	0	0	1	0	0
Milk in drinks	1 small glass (200 mL)	1	0	0	0	0	0
Total portions a day		**3 portions**	**6 portions**	**4 portions**	**5 portions**	**2** **portions**	**1 portion**

#### Physical activity advice

The programme also asks participants to meet physical activity recommendations for health (≥150 min of moderate intensity/week and resistance exercise two times per week) in line with the WHO guidelines on physical activity and sedentary behaviour.^26^ Suitability and safety to undertake an unsupervised home-based physical activity programme will be assessed using the Physical Activity Readiness questionnaire.^27^ Participation in physical activity is encouraged but is not essential for joining the trial. General practitioner clearance will be requested where required.

#### Dietetic support

Participants will receive their initial advice to follow the diet and engage in physical activity by phone or video call after completion of the 7 days of habitual diet records and ACTigraph physical activity monitoring (week 0). They will be offered dietetic support via phone or video call (MS teams) or email reviews with one of the trial dietitians at week 1, 4, 8, 12 after starting the programme and receive exit diet and physical activity advice after their 16-week review. Trial dietitians will have previous experience and/or recent training in breast cancer risk estimation, risk factors and advising intermittent diets.

Participants will also be provided with access to a trial specific private monitored Facebook group. This includes dietitian-led online group question and answer sessions, an opportunity to raise questions and have peer support chat with other women. Also updates on information on diet, the trial and risk factors for breast cancer risk. Access to the private Facebook group is by invite only to trial participants who are reminded not to post personal information. Participants who opt to use it will be advised of the data protection policy. At the end of the trial, all participants will be removed from the private Facebook group and the site will no longer be monitored.

### Outcomes

#### Co-primary feasibility outcomes

- Uptake rate as a percentage of the number of eligible participants invited who consent to take part for the trial.
- Retention rate measured as the number of consented participants who complete the 16-week trial.
- Self-reported adherence to the 6:1 programme across the 16-week trial.
- Patient acceptability of the 6:1 programme using a validated intervention evaluation questionnaire at the end of the programme or at time of withdrawal from the trial.^28^

#### Other outcomes include

- Key characteristics of those recruited versus the overall population in this age group in the main recruiting regional FHC.
- Key characteristics of those recruited who withdraw versus those who complete the trial.
- Fidelity of delivery and participant engagement with the programme: dietitian time required for delivery of the programme and participation engagement with the dietitian calls and the private 6:1 trial Facebook group. There are no plans to collate qualitative comments from the Facebook group.
- Preliminary evaluation of any benefits:
  - Changes in body weight and body composition; body fat and fat free mass (assessed with bioelectrical impedance), waist and hip circumferences.
  - Changes in diet quality using the Mediterranean diet score.^29^
  - Changes in alcohol intake and smoking using 7-day recall.^30^
  - Changes in physical activity using the International Physical Activity Questionnaire (IPAQ) short form.^31^
  - Change in objectively measured physical activity assessed with an ACTigraph.
- Preliminary evaluation of any possible harms:
  - Changes in eating behaviours using the BES.^22^
  - Change in sleep quality assessed using the PSQI.^32^
  - Potentially diet related adverse events including fatigue, constipation, dizziness, headache, indigestion in the 4 weeks before starting the diet and four weekly throughout the programme at each dietitian review call as defined by Common Terminology Criteria for Adverse Events (CTCAE) V.5.^33^
  - Change in mean menstrual cycle length during the trial from self-reported diaries.

#### Exploratory outcomes

We will explore the impact of low-calorie days on dietary intake on the remaining normal eating days of the week from the 7-day food record. Physical activity on low-calorie days and normal eating days from the 7 days using an ACTi graph activity monitor. Also, any differences in adherence to the low-calorie day across the menstrual cycle.

### Trial follow-up

**Figure 1:**
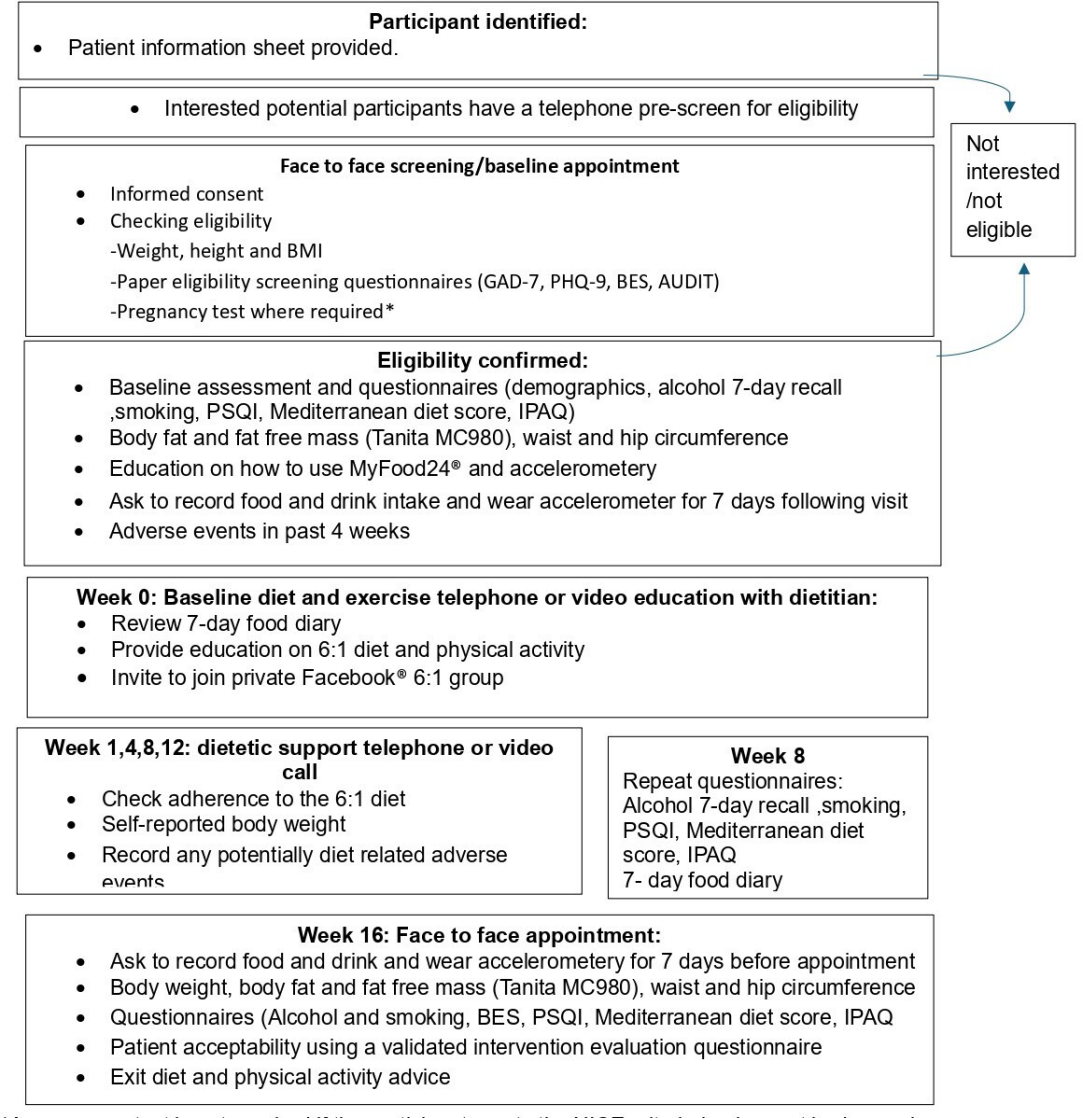
Study flow of participants through the Prevent 6:1 Trial. *A pregnancy test is not required if the participant meets the NICE criteria, that is, has not had sexual intercourse since the start of last normal menses or has been correctly and consistently using a reliable method of contraception. AUDIT, Alcohol Use Disorders Identification Test; BES, Binge Eating Scale30; GAD-7, General Anxiety Disorder-7; IPAQ, International Physical Activity Questionnaire; PHQ-9, Patient Health Questionnaire-9; PSQI, Pittsburgh Sleep Quality Index; NICE, National Institute of Clincial Excellence.

Participant flow through the trial is demonstrated in figure 1.

### Measurements

The full schedule of assessments is described in table 2.

**Table 2:** Schedule of assessments for participants in the Prevent 6:1 Trial

Procedures	Screening/baseline	Week 0	Week 4 (±7 days)	Week 8 (±7 days)	Week 12 (±7 days)	Week 16 (±7 days)
Informed consent	x					
Screening questionnaires GAD (anxiety), PHQ (depression), AUDIT (alcohol dependence)	x					
BES (Binge Eating Scale)	x			x		X
Pregnancy test where required*	x					
Medical history	x					
PAR-Q readiness to exercise questionnaire	x					
Demographic/dieting history questionnaire	x			Update any changes		Update any changes
Assessment of menstrual cycle dates across the trial			x	x	x	x
Adherence to the low-calorie day each week across the trial			x	x	x	x
Body weight, body fat and lean body mass (bioelectrical impedance), waist/hip circumference	x					x
Mediterranean diet questionnaire	x			x		x
7-day food diary on Myfood 24 or paper diary	Completed week after baseline visit			x		Complete in week before week 16 visit
Alcohol intake (7-day recall), smoking and vaping questionnaire	x			x		x
Wear ACTigraph physical activity monitor for 7 days	Complete in week after baseline visit					Complete in week before week 16 visit
IPAQ short form (physical activity)	x			x		x
PSQI (sleep quality)	x			x		x
Intervention evaluation questionnaire and self-reported acceptability data						x
Baseline and exit face-to-face diet and physical activity advice		x				x
Dietitian review phone or video call/email			Offered dietetic support via phone or video call (MS teams) or email reviews at weeks 1, 4, 8, 12			
Assessment of dietitian time spent delivering the intervention			x	x	x	x
Assessment of engagement with dietitian calls/emails/online sessions and private Facebook group			x	x	x	x
Recording potentially diet related adverse events in the previous 4 weeks	x		x	x	x	x
Home weight recording and reporting to dietitian			x	x	x	
Optional anonymous NIHR demographic questionnaire	x					

* Pregnancy test is not required if the participant meets NICE criteria for excluding pregnancy (negative predictive value of 99%–100%) (https://cks.nice.org.UnitedKingdom/topics/contraception-assessment/management/assessment-for-contraception/): Is within the first 5 days of the onset of a normal (natural) menstrual period. Has not had sexual intercourse since the start of last normal menses. Has been correctly and consistently using a reliable method of contraception.

AUDIT, Alcohol Use Disorders Identification Test; GAD-7, General Anxiety Disorder-7; IPAQ, International Physical Activity Questionnaire; NICE, National Institute of Clinical Excellence ; NIHR, National Institute of Health Research; PAR-Q, Physical Activity Readiness questionnaire; PHQ-9, Patient Health Questionnaire-9; PSQI, Pittsburgh Sleep Quality Index.

### Physical measurements

Body weight, body fat and fat-free mass will be assessed using bioelectrical impedance Tanita MC980. Participants will be asked to avoid food and drink for 90 min prior to this measurement.^34^ Waist and hip circumferences will be assessed using standardised methods.^35^

### Assessment of dietary intake

Participants will be asked to record 7-day dietary records using the MyFood 24 app or a paper food diary for a week following their baseline appointment before starting the 6:1 diet programme, and at weeks 8 and 16 in the programme. Participants will receive support from the research dietitians and written information on setting up and completing the MyFood 24 diary.

### Assessment of physical activity

Physical activity will be assessed for 1 week following their baseline appointment and the week before their 16-week review using a wrist worn accelerometry monitor (ACTigraph GT9X). Participants will be asked to wear the monitor for as much of this period as possible. Monitoring days will be included if wear time is greater than 10 hours/day.^36^ Baseline and 4-month activity data will be included when it includes at least three valid monitoring days with at least one of those days being a weekend day. We will assess time spent in sedentary, light, moderate, vigorous activity and total time in moderate vigorous physical activity. Also step count and vector magnitude average counts to get a measure of overall activity.^37^ Data will be analysed using ACTi Life 6 Software.

### Questionnaires

Participants will be asked to complete four paper questionnaires at the baseline visit to check eligibility; GAD-7,^23^ PHQ-9,^24^ BES,^22^ AUDIT.^38^ Eligible recruited participants will be asked to complete further questionnaires at baseline, weeks 8 and 16 (alcohol intake and smoking using 7-day recall,^30^ PSQI^32^ Mediterranean diet score,^29^ IPAQ^31^ using a secure web application (REDCap) (figure 1).^39^ A trial demographic questionnaire will also be requested at baseline. They will also be asked to complete an optional anonymous National Institute of Health Research (NIHR) demographic questionnaire. The NIHR is funding this project and they would like to understand whether research is reaching under-represented groups to help shape future research. Participants are informed their responses to questions are anonymous (not linked to their name or another identifier) and may be used for other educational and research purposes in the future.

Participants will also be asked to complete a trial specific diary over the 16-week programme, which records adherence to the low-calorie day of the 6:1 diet for each week and first day of each menstrual cycle while on the trial.

### Data analysis plan

Descriptive and basic statistics will be reported (number, frequencies and percentages and median and IQR as appropriate) as well as the level of missing data. Descriptive data for energy and macronutrient intake and physical activity will be assessed on the low-calorie days and the remaining days of the week to explore if the 6:1 intermittent diet has a carryover effect across the week.

Adherence to the 6:1 intermittent diet across the weeks of the menstrual cycle will inform any effects that the menstrual cycle has on adherence.

### Adverse events

Potentially diet related adverse events, that is, constipation, diarrhoea, dizziness, fatigue, headache, indigestion will be recorded retrospectively for the 4 weeks prior to baseline and prospectively every 4 weeks throughout the trial during the dietitian reviews using CTCAE V.5.0. Any grade 3 or grade 4 adverse events will be discussed with the trial management group. We will report any serious adverse events (SAEs) to the sponsor within 24 hours of the chief investigator becoming aware of the event. SAEs that are related and unexpected will be reported to the research ethics committee within 15 days of the chief investigator becoming aware of the event.

### Withdrawal of patients/stopping rules

Possible reasons for withdrawal of participants include:

1. Pregnancy during the trial.
2. Following safety review by the trial management group (TMG).
3. Increased level of risk of deterioration of mental state.
4. Unplanned loss of contact for ≥8 weeks.
5. Development of medical condition where weight control and diet and physical activity are no longer feasible or appropriate.
6. Participant choice to withdraw.

### Risks and burdens to participants

Participants may experience modest weight loss with the diet. We are minimising the risk for participants to become underweight (BMI <18.5 kg/m^2^) by recruiting women with a BMI of >20 kg/m^2^. We are asking participants to check and report their weight every 4 weeks during the trial to check they are not experiencing unexpected rapid weight loss due to over-restriction with diet. Likewise, we are minimising the risk of exacerbating existing disordered eating by excluding people with a history of eating disorders and high scores for binge-eating, anxiety and depression.

The binge eating and anxiety and depression questionnaires at baseline and during the trial and discussions with the trial dietitians may highlight psychological issues. Potential participants who are excluded from the trial at baseline due to high psychological scores will be provided guidance for who they can contact for further support and advised to self-refer to relevant support services including NHS Talking Therapies.

Those identified with moderate psychology scores, that is, PHQ-9 score of 10–14 (moderate depression), GAD-7 score of 10–14 (moderate anxiety), BES scores of 18–26 (moderate binge-eating), AUDIT-C scores of 8–15 (increasing risk of drinking disorder) will be asked the history of this and whether they are currently receiving support/treatment and what this entails. Those not currently receiving treatment will be provided guidance for who they can contact for further support and signposted to appropriate local services and we will seek permission to copy this information to their general practitioner.

Trial questionnaires completed at baseline and during the trial will be reviewed weekly during the trial and participants contacted and triaged to the appropriate services as outlined above.

### Data management

Participant data will be anonymised and stored and archived in line with the Data Protection Act (2018). The sponsor will periodically audit the site trial file, a sample of the case report form, consent forms and source data, and check accuracy of the trial database to ensure satisfactory completion. Data will be collected and managed using REDCap^39^ (Vanderbilt University, United States) an electronic data capture tool hosted at MFT. This included electronic questionnaires and case report forms. Dietary intake data will be collected online using my Food 24. ACTigraph data will be downloaded using ACTi Life 6 Software. Any paper questionnaires and food diaries received will be stored securely in a locked filing cabinet in the research dietitian’s office. Engagement with the private Facebook will be downloaded from the private Facebook page.

### Trial management

The TMG comprises the chief investigator (MHar), a statistician (EB), trial manager (NJ), research dietitians (CL, MHai), clinical lead for the FHC (SH), expertise in behaviour change, psychology and well-being (DF). Also, two public contributors (KC, ES). All aspects of the trial and trial personnel will adhere to the trial protocol (V.1.2 or subsequent approved versions) and Good Clinical Practice and Data Protection principles. Three monthly TMG meetings will review trial processes, recruitment and retention, data collection, safety information and grade 3 or 4 adverse events.

### Patient and public involvement

Previous work from our group highlighted an unmet need for a weight gain prevention intervention among young women.^40^ Two multi-ethnic patient public and involvement discussion groups (November 2022 and March 2023) supported a trial to test a 6:1 programme rather than other possible dietary approaches including time-restricted eating. This session also informed methods of recruitment and the types of resources required.

Two public contributors have been recruited for the duration of the project. They have reviewed the trial design, the patient burden of participating in the trial and patient materials. They will continue to work with the research team to help collate free text feedback from participants from the acceptability questionnaire and to advise on best routes of research dissemination to reach the target audience.

## Ethics and dissemination

This trial has been approved by the South Central—Berkshire B Research Ethics committee (rec reference 24/SC/0321) and is sponsored by MFT. All ethical amendments and documentation will be approved by ethics and the research sponsor.^17^

### Implications and future directions

Success of this feasibility trial will be defined by the progression criteria described in table 3. If these criteria are met, funding will be sought for a future efficacy trial.

**Table 3:** Progression criteria to an efficacy trial of the 6:1 diet intervention

Progression criteria	Feasible (green)	Feasible with modification of the protocol (amber)	Not feasible (red)
Recruitment	≥6 patients/month	3–5 patients/month	≤2 patients/month
Uptake to the feasibility trial	>15%	10%–15%	<10%
Retention to the feasibility trial	>70%	50%–70%	<50%
Adherence to the ILED diet: % of the low-calorie days completed (1 day/week for 16 weeks)	>50%	30%–50%	<30%
Safety concerns: evidence of increased binge eating/reduced sleep/reduced physical activity/reduced diet quality	Reported in <10% of participants	Reported in 10%–20% of participants	Reported in >20% of participants

ILED, Intermittent Low Energy Diet.

### Dissemination

Findings will be disseminated via peer-reviewed journals, national and international cancer prevention and obesity meetings and conferences and cancer prevention charitable bodies and organisations in the UK, such as Prevent Breast Cancer (Registered Charity 1109839).

### Data sharing

The protocol and data analysis plan is available from the author on reasonable request. At the end of the trial an anonymised dataset will be uploaded onto a data sharing repository, that is, Fig share in accordance with FAIR principles for data sharing.

## Supplementary material

10.1136/bmjopen-2025-113204 online supplemental file 1
