# Supplementary material for: Prevent 6:1 trial: pI rotocol for a feasibility trial of a 6:1 intermittent diet for weight gain prevention in women at increased risk of breast cancer
Source: BMJ Open. 2026 Apr 29;16(4):e113204. doi: 10.1136/bmjopen-2025-113204 (PMC13140951; doi:10.1136/bmjopen-2025-113204)
Supplement: online supplemental file 1 [file bmjopen-16-4-s001.docx]

**Research Dietitians**

**Michelle Harvie**

**Cheryl Lombardelli**

**Mai Haibi**

**Tel:**

**Email:**

Research Dietitians

1^st^ Floor Education Research Centre

Manchester University NHS Foundation Trust

Wythenshawe Hospital

Manchester

M23 9LT

Title of Project: The 6:1 Diet for the reduction of risk of Breast Cancer Study

IRAS ID: 343256

Study Number: B02253

Participant Identification Number for this trial __________

Name of Researcher Professor Michelle Harvie

# **Participant Consent Form**

**Please initial box**

| 1. I confirm that I have read and understand the participant information sheet (version 1 date 18.9.24) for the above study. I have had the opportunity to consider the information and ask questions, and have had these answered satisfactorily. |
| --- |
| 1. I understand that my participation is voluntary and that I am free to withdraw at any time, without giving any reason, without my medical care or legal rights being affected. |
| 1. I understand that relevant sections of my medical notes and data collected during the study may be looked at by individuals from Manchester University NHS Foundation Trust and regulatory authorities, where it is relevant to my taking part in the research. I give permission for these individuals to have access to my records. |
| 1. I understand that the information collected about me will be used to support other research in the future and may be shared anonymously with other researchers. |
|  |
| 1. I agree to my GP being informed of my participation in this study and changes to my weight, body measurements and any abnormal findings on questionnaires. |
| 1. I have informed the study team of any health issues, including those which may affect my ability to follow the diet or be physically active. I will inform the study team of changes to my health status during the study. 2. I understand the effects of the diet during pregnancy are not known and I agree to have a pregnancy test at the initial appointment where there is a possibility I could be pregnant. 3. I understand the effects of the diet during pregnancy are not known and I should avoid getting pregnant during the study by using effective contraception or abstinence (no sex). 4. I agree to wear a special watch for 7 days at the start and end of the study which counts my steps and the amount of time I have spent at rest, doing light, or moderate or vigorous intensity exercise (Actigraph®). 5. I consent to the storage of personal information (including electronic) for the purposes of this study. I understand that any information that could identify me will be kept strictly confidential and that no personal information will be included in the study report or other publication. 6. I agree that relevant information about my healthcare can be obtained from my medical records within the 12 month study duration if I withdraw from the study early. 7. I agree to take part in the above study. |
|  |
|  |
|  |
|  |
|  |

**Optional** **Questions** (you can still participate in the study if you do not wish to use the MyFood24 App or Facebook).

1. I understand if I opt to use the MyFood24 App that I need to provide my personal email, and I consent for this to be used in the App.
2. I understand that information I provide to MyFood24 is stored on secure servers in Ireland and that I can request for any of my data to be removed at any time.
3. I understand if I opt to join the study’s private Facebook Group I will need to provide my personal email, and I consent for this to be used for that feature.
4. I have read, understood and I agree to follow the study’s private Facebook Group ground rules.
5. I understand that information I provide to the study’s private Facebook Group is stored on secure servers in Europe and the USA and that I can request for any of my data to be removed at any time.
6. I would like to receive a summary of the final study results Yes  No

…………………………..………... ………………….. …………..………………

Name of participant Date Signature

……………………………..……... …………………. ……………..……………

Name of person taking consent Date Signature
