# Supplementary material for: Prevent 6:1 trial: pI rotocol for a feasibility trial of a 6:1 intermittent diet for weight gain prevention in women at increased risk of breast cancer
Source: BMJ Open. 2026 Apr 29;16(4):e113204. doi: 10.1136/bmjopen-2025-113204 (PMC13140951; doi:10.1136/bmjopen-2025-113204)
Supplement: Supplementary file 2 [file bmjopen-2025-113204.reviewer_comments.pdf]

PEER REVIEW HISTORY

BMJ Open publishes all reviews undertaken for accepted manuscripts. Reviewers are asked to complete a checklist review form and are provided with free text boxes to elaborate on their assessment. These free text comments are reproduced below.

ARTICLE DETAILS

Title (Provisional)

The Prevent 6:1 Trial -Protocol for a feasibility trial of a 6:1 intermittent diet for weight gain prevention in women at increased risk of breast cancer

Authors

Kinsey, Laura; French, David; Howell, Sacha; Gill, Jason; Lombardelli, Cheryl; Haiba, Mai; Barrett, Emma; Jones, Natasha; Wisely, Julie; Harvie, Michelle

---

VERSION 1 - REVIEW

**Reviewer**	**1**
**Name**	**Carson, Tiffany**
**Affiliation**	**Moffitt Cancer Center**
**Date**	**11-Feb-2026**
**COI**	**None**

Thank you for the opportunity to review this manuscript. The authors describe the rationale and design of a single-arm trial (n=30) to assess the feasibility of a 6:1 intermittent fasting diet program to promote healthy eating and prevent weight gain among women at increased risk of breast cancer with a body mass index of 20-25 kg/m2. Overall, this is a well written manuscript with many strengths that I believe would be of interest to BMJ open readers. I have a few minor comments, which I hope will strengthen the manuscript.

Introduction

1. Can the authors provide national statistics that would help frame the potential scope and impact of the planned work? For example, what is the prevalence women with normal weight, overweight, and obesity nationally.

Methods and Analysis

2. Please clarify whether screening and data collection visits will take place at the FHCs or a centralized institutional location.

3. In Table 1, it states that participants need to be able to communicate in “English or any other language”. Is that correct? Can all languages be included?

4. Please provide more details about the “run-in week”. It is mentioned but the rationale/purpose and planned activities, if any, are unclear.

5. Will any data (e.g., utilization, qualitative notes) be captured from the private Facebook group?

6. Please expand the description of the methods that will be used for physical measurements and wearing the actigraph.

7. Please include the anticipated dates of the study in the manuscript.

Minor Typographical

Page 4, Line 56: “physical interventions” should be “physical activity interventions”

Page 17, Line 19: Please expand the abbreviation “GP”.

VERSION 1 - AUTHOR RESPONSE

Reviewer: 1

Dr. Tiffany Carson, Moffitt Cancer Center

Comments to the Author:

Thank you for the opportunity to review this manuscript. The authors describe the rationale and design of a single-arm trial (n=30) to assess the feasibility of a 6:1 intermittent fasting diet program to promote healthy eating and prevent weight gain among women at increased risk of breast cancer with a body mass index of 20-25 kg/m2. Overall, this is a well written manuscript with many strengths that I believe would be of interest to BMJ open readers. I have a few minor comments, which I hope will strengthen the manuscript.

Introduction

1. Can the authors provide national statistics that would help frame the potential scope and impact of the planned work? For example, what is the prevalence women with normal weight, overweight, and obesity nationally.

We have now added the national statistics in the introduction on page 3

In the UK 35% of women are a healthy weight with 62% overweight or obese

Methods and Analysis

2. Please clarify whether screening and data collection visits will take place at the FHCs or a centralized institutional location.

This has been clarified on page 3 in the trial setting and recruitment section.

All trial face to face assessments will be undertaken by the research team in a research facility at the main recruiting site at Wythenshawe Hospital.

3. In Table 1, it states that participants need to be able to communicate in "English or any other language". Is that correct? Can all languages be included?

This has been clarified to say:

Able to communicate (written and spoken) in English or any another language and access to a phone as we have access to an interpreter service

4. Please provide more details about the "run-in week". It is mentioned but the rationale/purpose and planned activities, if any, are unclear.

This has been clarified on page 5/6.

All participants will have a trial run- in week before they receive baseline diet advice when we ask them to record 7 days of habitual diet and wear an activity monitor to assess their habitual diet and physical activity patterns

5. Will any data (e.g., utilization, qualitative notes) be captured from the private Facebook group?

Ther are no plans for this which is clarified on page 11

There are no plans to collate qualitative comments from the Facebook® group

6. Please expand the description of the methods that will be used for physical measurements and wearing the actigraph.

This is a good point and now described in more detail in the methods section on page 15

Participants will be asked to wear the monitor for as much of this period as possible. Monitoring days will be included if wear time is greater than 10 hours/ day (36). Baseline and 4-month activity data will be included when it includes at least three valid monitoring days with at least one of those days being a weekend day. We will assess time spent in sedentary, light, moderate, vigorous activity and total time in moderate vigorous physical activity. Also step count and vector magnitude average counts to get a measure of overall activity (37).Data will be analysed using ACTi Life 6 Software.

7. Please include the anticipated dates of the study in the manuscript.

These have been included on page 5

The planned trial start and end dates are 17th March 2025 and 17th March 2026.

Minor Typographical

Page 4, Line 56: “physical interventions” should be “physical activity interventions”

This has been corrected on page 3

Page 17, Line 19: Please expand the abbreviation “GP”.

This has been expanded to General practitioner on page 17
